# Supplementary material for: Variations in the Second Derivative of a Photoplethysmogram with Age in Healthy Korean Adults
Source: Int J Environ Res Public Health. 2022 Dec 23;20(1):236. doi: 10.3390/ijerph20010236 (PMC9819370; doi:10.3390/ijerph20010236)
Supplement: Supplementary file 1 [file ijerph-20-00236-s001.zip › ijerph-1984006-supplementary.pdf]

Table S1. The data on healthy Koreans' SDPTG indices.

Subject	Gender (1=m,2=f)	Age	b/a	c/a	d/a	e/a	SDPTG-AI
m-19-lxm214	1	19	-0.7805	0.0421	0.1403	0.3034	-1.2664
m-21-lxm229	1	21	-0.7812	0.0435	-0.0721	0.2033	-0.9558
m-21-lxm227	1	21	-0.7145	0.0172	-0.0479	0.1773	-0.8612
m-22-lxm220	1	22	-0.7262	0.0491	-0.0281	0.1908	-0.9380
m-22-lxm205	1	22	-0.7059	-0.0004	-0.1870	0.2166	-0.7351
m-22-lxm201	1	22	-0.8158	0.1586	-0.0777	0.1078	-1.0045
m-22-lxm224	1	22	-0.5695	0.0192	-0.0490	0.2059	-0.7455
m-23-lxm222	1	23	-0.6830	0.0768	-0.0856	0.0355	-0.7097
m-23-lxm210	1	23	-0.7656	-0.0635	-0.0016	0.1123	-0.8128
m-23-lxm209	1	23	-0.7409	0.0340	-0.1707	0.1345	-0.7387
m-23-lxm225	1	23	-0.6355	0.0489	0.0929	0.2468	-1.0242
m-23-lxm219	1	23	-0.8257	0.0513	-0.0859	0.0968	-0.8879
m-23-lxm230	1	23	-0.6849	-0.2329	-0.1233	0.3151	-0.6438
m-24-lxm206	1	24	-0.9018	-0.0347	-0.0741	0.2139	-1.0069
m-24-lxm204	1	24	-0.6765	-0.2500	-0.1471	0.1176	-0.3971
m-24-lxm221	1	24	-0.7392	-0.0050	0.0972	0.3473	-1.1788
m-24-lxm215	1	24	-0.8246	-0.2871	-0.0608	0.4441	-0.9208
m-25-lxm211	1	25	-0.6650	0.0431	-0.0954	0.1314	-0.7441
m-25-lxm212	1	25	-0.9538	-0.0003	-0.0169	0.3333	-1.2698
m-25-lxm213	1	25	-0.7056	-0.1083	-0.0625	0.1972	-0.7319
m-25-lxm207	1	25	-0.7073	-0.0774	-0.1100	0.1655	-0.6853
m-25-lxm202	1	25	-0.6954	0.0703	-0.1003	0.1250	-0.7904
m-26-lxm217	1	26	-0.7025	0.0466	-0.2038	0.3823	-0.9276
m-27-lxm226	1	27	-0.7356	0.0637	0.0062	0.1546	-0.9601
m-27-lxm228	1	27	-0.6354	-0.0270	-0.1925	0.1243	-0.5402
m-27-lxm223	1	27	-0.6909	0.0075	-0.0256	0.2347	-0.9075
m-28-lxm208	1	28	-0.7118	0.0680	-0.1509	0.1458	-0.7746
m-28-lxm203	1	28	-0.7680	-0.0282	-0.1291	0.1243	-0.7350
m-29-lxm218	1	29	-0.7632	0.0727	-0.1677	0.1283	-0.7965
m-29-lxm216	1	29	-0.8044	0.0174	-0.0363	0.3125	-1.0979
m-30-lxm326	1	30	-0.6739	-0.2266	-0.0664	0.2929	-0.6739
m-31-lxm320	1	31	-0.7259	0.1331	-0.2774	0.0835	-0.6651
m-31-lxm312	1	31	-0.6897	0.0629	-0.2713	0.1728	-0.6540
m-31-lxm306	1	31	-0.7077	-0.1185	-0.0954	0.2615	-0.7554
m-32-lxm318	1	32	-0.5304	-0.1243	-0.2135	0.2402	-0.4329
m-33-lxm324	1	33	-0.6887	0.0191	-0.1318	0.0991	-0.6752
m-33-lxm316	1	33	-0.9048	-0.2843	-0.2121	0.3362	-0.7446
m-33-lxm323	1	33	-0.8471	0.0194	-0.1459	0.1971	-0.9176
m-33-lxm311	1	33	-0.7614	0.0678	-0.1629	0.0549	-0.7211
m-33-lxm301	1	33	-0.7494	0.0405	-0.1535	0.0919	-0.7282

m-34-lxm309	1	34	-0.5082	-0.0391	-0.1689	0.1782	-0.4784
m-34-lxm302	1	34	-0.8813	-0.1396	-0.1753	0.1549	-0.7212
m-34-lxm307	1	34	-0.6919	0.1559	-0.2938	0.1339	-0.6880
m-34-lxm317	1	34	-0.8253	0.1192	-0.3140	0.1676	-0.7981
m-36-lxm313	1	36	-0.7240	0.0878	0.0218	0.3033	-1.1370
m-36-lxm325	1	36	-0.6092	0.0211	-0.2726	0.1386	-0.4963
m-37-lxm319	1	37	-0.5431	-0.0241	-0.0485	0.1728	-0.6434
m-37-lxm321	1	37	-0.6652	0.0035	-0.1995	0.1272	-0.5965
m-37-lxm305	1	37	-0.9536	0.1096	-0.2515	0.1271	-0.9389
m-37-lxm314	1	37	-0.7019	0.3399	-0.0738	0.0801	-1.0480
m-37-lxm328	1	37	-0.9027	0.0480	-0.0653	0.1516	-1.0370
m-37-lxm304	1	37	-0.6563	-0.2969	-0.2500	0.1406	-0.2500
m-37-lxm308	1	37	-0.6145	-0.0736	-0.1619	0.1200	-0.4991
m-37-lxm322	1	37	-0.7358	-0.0072	-0.0795	0.1513	-0.8003
m-37-lxm330	1	37	-0.7039	-0.0644	-0.2204	0.1738	-0.5928
m-38-lxm310	1	38	-0.7914	0.0271	-0.0703	0.2017	-0.9499
m-38-lxm303	1	38	-0.7608	0.0393	-0.1560	0.1508	-0.7949
m-39-lxm315	1	39	-0.8456	0.1107	-0.2225	0.2795	-1.0134
m-39-lxm329	1	39	-0.9114	-0.0439	-0.0464	0.1595	-0.9805
m-39-lxm327	1	39	-0.7459	-0.1148	-0.2681	0.2384	-0.6013
m-40-lxm412	1	40	-0.7245	-0.1431	-0.2459	0.2374	-0.5728
m-40-lxm411	1	40	-0.6305	-0.0523	-0.2687	0.1659	-0.4754
m-41-lxm417	1	41	-0.7063	-0.1083	-0.3437	0.1749	-0.4292
m-41-lxm409	1	41	-0.6962	-0.1761	-0.2747	0.2442	-0.4896
m-41-lxm415	1	41	-0.6778	-0.1220	-0.2643	0.2196	-0.5112
m-42-lxm408	1	42	-0.6618	-0.2647	-0.2059	0.2500	-0.4412
m-42-lxm405	1	42	-0.8742	0.0269	-0.0873	0.1766	-0.9905
m-42-lxm401	1	42	-0.7142	-0.0166	-0.3095	0.1175	-0.5056
m-42-lxm430	1	42	-0.9061	0.0659	-0.1910	0.2190	-1.0001
m-42-lxm422	1	42	-0.6926	-0.1531	-0.2543	0.1570	-0.4422
m-43-lxm402	1	43	-0.5724	-0.1004	-0.1806	0.1489	-0.4404
m-43-lxm426	1	43	-0.6918	-0.0687	-0.3205	0.1230	-0.4257
m-44-lxm423	1	44	-0.5244	-0.2046	-0.3596	0.1520	-0.1122
m-44-lxm429	1	44	-0.5151	-0.1597	-0.3080	0.1295	-0.1769
m-44-lxm414	1	44	-0.7317	-0.0588	-0.1680	0.2994	-0.8044
m-45-lxm428	1	45	-0.7345	-0.0174	-0.3335	0.2084	-0.5921
m-45-lxm425	1	45	-0.6351	0.0109	-0.3367	0.1756	-0.4849
m-46-lxm420	1	46	-0.6529	0.0008	-0.2242	0.1356	-0.5651
m-46-lxm520	1	46	-0.7711	-0.0945	-0.1656	0.1463	-0.6573
m-47-lxm427	1	47	-0.6982	0.1353	-0.2937	0.1402	-0.6800
m-47-lxm418	1	47	-0.5217	-0.0207	-0.1783	0.1344	-0.4570
m-48-lxm407	1	48	-0.6635	-0.1117	-0.1399	0.1831	-0.5949
m-48-lxm421	1	48	-0.5708	-0.1562	-0.1923	0.2100	-0.4323

m-49-lxm416	1	49	-0.6981	-0.0264	-0.2152	0.2114	-0.6679
m-49-lxm419	1	49	-0.6295	-0.1573	-0.2679	0.1261	-0.3304
m-49-lxm403	1	49	-0.7762	-0.0930	-0.1923	0.2771	-0.7681
m-49-lxm424	1	49	-0.7382	-0.0308	-0.2129	0.1142	-0.6087
m-50-lxm516	1	50	-0.6747	-0.0396	-0.2751	0.0927	-0.4527
m-50-lxm522	1	50	-0.5098	-0.1583	-0.3650	0.1660	-0.1526
m-51-lxm531	1	51	-0.5880	0.0448	-0.4255	0.1977	-0.4050
m-51-lxm526	1	51	-0.5333	-0.2124	-0.3257	0.2743	-0.2695
m-52-lxm519	1	52	-0.5712	-0.2017	-0.3480	0.2731	-0.2946
m-52-lxm503	1	52	-0.5413	-0.2591	-0.3154	0.2046	-0.1715
m-52-lxm510	1	52	-0.5162	-0.0272	-0.4005	0.1267	-0.2152
m-52-lxm518	1	52	-0.5348	-0.1647	-0.1927	0.2338	-0.4112
m-52-lxm517	1	52	-0.4855	-0.3155	-0.4693	0.2925	0.0069
m-52-lxm513	1	52	-0.6775	-0.1950	-0.2232	0.2619	-0.5213
m-53-lxm528	1	53	-0.4121	-0.1847	-0.4121	0.1136	0.0711
m-53-lxm529	1	53	-0.5995	-0.1050	-0.2317	0.0924	-0.3552
m-54-lxm505	1	54	-0.3810	-0.1460	-0.2376	0.1387	-0.1360
m-54-lxm511	1	54	-0.6808	-0.0857	-0.1986	0.1795	-0.5759
m-54-lxm502	1	54	-0.5911	-0.0403	-0.2813	0.1529	-0.4224
m-54-lxm512	1	54	-0.4164	-0.2162	-0.3045	0.0986	0.0057
m-55-lxm523	1	55	-0.5170	-0.2079	-0.3536	0.1947	-0.1502
m-55-lxm509	1	55	-0.6013	-0.1967	-0.2600	0.1640	-0.3087
m-55-lxm514	1	55	-0.4520	-0.0892	-0.4087	0.1377	-0.0919
m-55-lxm506	1	55	-0.7373	-0.2988	-0.3093	0.2571	-0.3863
m-56-lxm524	1	56	-0.5505	0.0013	-0.3775	0.2745	-0.4488
m-56-lxm508	1	56	-0.4983	-0.2040	-0.2999	0.2094	-0.2038
m-56-lxm527	1	56	-0.4689	-0.2576	-0.4689	0.1402	0.1174
m-57-lxm521	1	57	-0.5246	-0.2726	-0.4388	0.1557	0.0311
m-57-lxm525	1	57	-0.5593	-0.2525	-0.3563	0.3536	-0.3042
m-58-lxm530	1	58	-0.7307	-0.2410	-0.3315	0.1275	-0.2856
m-58-lxm501	1	58	-0.4214	-0.1409	-0.2799	0.1286	-0.1292
m-59-lxm504	1	59	-0.5467	0.0064	-0.5467	0.1336	-0.1400
m-59-lxm507	1	59	-0.4663	-0.1665	-0.2713	0.1137	-0.1422
m-59-lxm515	1	59	-0.6160	-0.1152	-0.1682	0.1809	-0.5135
m-60-lxm606	1	60	-0.7589	-0.0872	-0.3355	0.2182	-0.5544
m-60-lxm629	1	60	-0.4777	-0.2106	-0.3283	0.2638	-0.2026
m-60-lxm626	1	60	-0.5282	-0.1987	-0.5282	0.2155	-0.0168
m-60-lxm622	1	60	-0.4543	-0.1187	-0.4520	0.1311	-0.0146
m-61-lxm618	1	61	-0.5014	-0.2687	-0.4399	0.2238	-0.0166
m-61-lxm601	1	61	-0.6304	-0.1815	-0.3301	0.1609	-0.2797
m-61-lxm610	1	61	-0.4994	-0.0860	-0.4994	0.2090	-0.1230
m-61-lxm607	1	61	-0.5614	-0.1847	-0.2494	0.0910	-0.2183
m-61-lxm613	1	61	-0.4659	-0.1028	-0.3788	0.1556	-0.1399

m-61-lxm611	1	61	-0.5458	-0.2670	-0.5458	0.0818	0.1852
m-62-lxm620	1	62	-0.4624	-0.2657	-0.3907	0.1367	0.0571
m-62-lxm605	1	62	-0.6629	0.0103	-0.1507	0.2795	-0.8020
m-62-lxm623	1	62	-0.5060	-0.2083	-0.4898	0.1994	-0.0072
m-62-lxm625	1	62	-0.5329	-0.1973	-0.2783	0.1080	-0.1653
m-63-lxm602	1	63	-0.6486	-0.2429	-0.1196	0.1019	-0.3880
m-63-lxm619	1	63	-0.6091	-0.1415	-0.1710	0.2071	-0.5037
m-63-lxm609	1	63	-0.5923	-0.2381	-0.2789	0.1943	-0.2697
m-63-lxm616	1	63	-0.6402	-0.1325	-0.2623	0.1533	-0.3986
m-64-lxm615	1	64	-0.5611	-0.0332	-0.3985	0.1030	-0.2324
m-64-lxm614	1	64	-0.5764	-0.1905	-0.2169	0.1015	-0.2706
m-64-lxm604	1	64	-0.5296	-0.1419	-0.3591	0.1067	-0.1353
m-65-lxm624	1	65	-0.6141	-0.1146	-0.3164	0.1462	-0.3293
m-65-lxm617	1	65	-0.6022	-0.1956	-0.2781	0.1138	-0.2423
m-65-lxm627	1	65	-0.7811	-0.0724	-0.2030	0.0881	-0.5938
m-65-lxm603	1	65	-0.6578	-0.0334	-0.2749	0.0300	-0.3796
m-66-lxm628	1	66	-0.3385	-0.2769	-0.5231	0.0615	0.4000
m-66-lxm621	1	66	-0.4724	-0.1189	-0.2975	0.1564	-0.2124
m-66-lxm630	1	66	-0.5879	-0.1568	-0.3393	0.1379	-0.2297
m-67-lxm612	1	67	-0.6690	-0.1637	-0.2101	0.1011	-0.3963
m-69-lxm608	1	69	-0.4787	-0.0987	-0.4153	0.0696	-0.0343
f-19-lxf204	2	19	-0.4186	-0.0064	-0.1802	0.0770	-0.3090
f-19-lxf205	2	19	-0.6306	0.0501	-0.0408	0.0604	-0.7002
f-19-lxf209	2	19	-0.7811	-0.0844	0.0127	0.2466	-0.9559
f-20-lxf220	2	20	-0.5674	-0.0610	-0.0782	0.2231	-0.6512
f-20-lxf219	2	20	-0.6996	0.0120	-0.1935	0.1458	-0.6639
f-20-lxf221	2	20	-0.5189	-0.0313	-0.2673	0.1808	-0.4010
f-20-lxf228	2	20	-0.8668	0.1774	-0.2393	0.1683	-0.9732
f-21-lxf203	2	21	-0.5608	0.0231	-0.1233	0.1346	-0.5951
f-21-lxf210	2	21	-0.6318	0.1283	-0.0630	0.2557	-0.9527
f-21-lxf231	2	21	-1.0067	0.2145	-0.2914	0.1574	-1.0873
f-21-lxf208	2	21	-0.7949	0.0836	-0.0503	0.0507	-0.8790
f-22-lxf212	2	22	-0.5504	0.0093	-0.0872	0.1577	-0.6303
f-22-lxf202	2	22	-0.7502	-0.0130	-0.0761	0.0982	-0.7593
f-23-lxf211	2	23	-0.5243	0.0038	-0.1628	0.1222	-0.4875
f-24-lxf226	2	24	-0.7978	0.0292	-0.2021	0.1126	-0.7376
f-24-lxf224	2	24	-0.5786	-0.0034	-0.0944	0.1092	-0.5900
f-24-lxf216	2	24	-0.5741	-0.0120	-0.1252	0.1296	-0.5665
f-25-lxf201	2	25	-0.6409	0.0337	-0.2187	0.0910	-0.5470
f-25-lxf227	2	25	-0.5579	-0.0371	-0.1022	0.1677	-0.5862
f-25-lxf206	2	25	-0.6268	0.0243	-0.0908	0.1507	-0.7110
f-25-lxf225	2	25	-0.7407	0.0105	-0.1460	0.0842	-0.6893
f-26-lxf230	2	26	-0.5791	0.0903	-0.0506	0.1949	-0.8137

f-27-lxf222	2	27	-0.6208	-0.0808	-0.0102	0.1472	-0.6770
f-27-lxf213	2	27	-0.5966	0.0203	-0.2513	0.1428	-0.5084
f-27-lxf229	2	27	-0.5140	0.0019	-0.1133	0.1523	-0.5549
f-28-lxf223	2	28	-0.7141	-0.0133	-0.1330	0.1029	-0.6707
f-28-lxf214	2	28	-0.5646	-0.0548	-0.0672	0.1639	-0.6065
f-29-lxf218	2	29	-0.5288	-0.0787	-0.2043	0.0774	-0.3232
f-29-lxf217	2	29	-0.6310	-0.0658	-0.0888	0.1185	-0.5948
f-30-lxf321	2	30	-0.5500	-0.0705	-0.1922	0.1062	-0.3935
f-31-lxf325	2	31	-0.4819	-0.0133	-0.1656	0.1029	-0.4060
f-31-lxf318	2	31	-0.5343	-0.0554	-0.2942	0.1291	-0.3138
f-31-lxf215	2	31	-0.5322	-0.1266	-0.2660	0.2259	-0.3655
f-31-lxf311	2	31	-0.7392	-0.0062	-0.1458	0.1496	-0.7368
f-31-lxf327	2	31	-0.4291	-0.0592	-0.1400	0.1599	-0.3898
f-32-lxf330	2	32	-0.5418	-0.0942	-0.1911	0.1758	-0.4322
f-32-lxf319	2	32	-0.5807	-0.0620	-0.2300	0.1567	-0.4454
f-33-lxf309	2	33	-0.5457	-0.0120	-0.0443	0.2290	-0.7184
f-33-lxf322	2	33	-0.6155	-0.0089	-0.1803	0.2744	-0.7006
f-34-lxf310	2	34	-0.5725	-0.0778	-0.1057	0.1368	-0.5258
f-34-lxf301	2	34	-0.5375	-0.0537	-0.2658	0.0627	-0.2806
f-35-lxf324	2	35	-0.6957	-0.0651	-0.2520	0.1546	-0.5332
f-36-lxf328	2	36	-0.5804	-0.0711	-0.2637	0.1403	-0.3859
f-36-lxf326	2	36	-0.7264	-0.2392	-0.2530	0.1487	-0.3829
f-36-lxf329	2	36	-0.4941	-0.1233	-0.2140	0.1156	-0.2722
f-37-lxf306	2	37	-0.7531	-0.0336	-0.1740	0.1513	-0.6967
f-37-lxf312	2	37	-0.7879	-0.0345	-0.1211	0.1612	-0.7936
f-37-lxf302	2	37	-0.6208	-0.0808	-0.0102	0.1472	-0.6770
f-37-lxf316	2	37	-0.4531	-0.1804	-0.3952	0.1490	-0.0265
f-37-lxf314	2	37	-0.6706	-0.1289	-0.1693	0.0771	-0.4495
f-38-lxf303	2	38	-0.6882	-0.1205	-0.1550	0.1264	-0.5391
f-38-lxf315	2	38	-0.3237	-0.1218	-0.2828	0.1132	-0.0322
f-38-lxf323	2	38	-0.5754	-0.0716	-0.1042	0.1106	-0.5103
f-38-lxf317	2	38	-0.5446	-0.0944	-0.2772	0.1653	-0.3383
f-38-lxf305	2	38	-0.7228	-0.0453	-0.1239	0.1494	-0.7029
f-39-lxf308	2	39	-0.5625	0.0230	-0.3132	0.1962	-0.4686
f-39-lxf304	2	39	-0.4303	-0.1975	-0.3284	0.0930	0.0026
f-39-lxf313	2	39	-0.6866	-0.0329	-0.1500	0.3067	-0.8104
f-40-lxf421	2	40	-0.5592	-0.1853	-0.1913	0.1343	-0.3170
f-40-lxf425	2	40	-0.5369	-0.0821	-0.1457	0.1613	-0.4704
f-41-lxf428	2	41	-0.4356	-0.1078	-0.3069	0.1313	-0.1522
f-41-lxf408	2	41	-0.3918	-0.1000	-0.3118	0.1120	-0.0920
f-41-lxf418	2	41	-0.4993	-0.1938	-0.2809	0.1097	-0.1343
f-42-lxf426	2	42	-0.4102	-0.0372	-0.2954	0.0969	-0.1746
f-42-lxf405	2	42	-0.6851	-0.0209	-0.3211	0.1683	-0.5114

f-42-lxf423	2	42	-0.6804	0.0004	-0.1506	0.1938	-0.7241
f-42-lxf422	2	42	-0.6451	-0.0254	-0.2135	0.1839	-0.5902
f-43-lxf411	2	43	-0.5132	-0.1308	-0.2221	0.1185	-0.2788
f-44-lxf412	2	44	-0.5656	-0.1624	-0.3968	0.1849	-0.1913
f-44-lxf416	2	44	-0.4004	-0.1161	-0.3867	0.1285	-0.0261
f-45-lxf413	2	45	-0.6015	-0.2088	-0.2788	0.2336	-0.3475
f-45-lxf427	2	45	-0.4233	-0.1391	-0.3549	0.1861	-0.1154
f-46-lxf406	2	46	-0.5921	-0.1930	-0.2258	0.2932	-0.4666
f-46-lxf420	2	46	-0.4274	-0.1790	-0.4142	0.1593	0.0067
f-46-lxf403	2	46	-0.5438	-0.1346	-0.2098	0.1605	-0.3599
f-46-lxf409	2	46	-0.5364	-0.2242	-0.3353	0.1871	-0.1641
f-47-lxf430	2	47	-0.8017	-0.0101	-0.1577	0.0781	-0.7120
f-47-lxf407	2	47	-0.4921	-0.1552	-0.2544	0.1704	-0.2529
f-47-lxf415	2	47	-0.4872	-0.2076	-0.3070	0.1342	-0.1068
f-47-lxf424	2	47	-0.6069	-0.0594	-0.1848	0.1117	-0.4743
f-48-lxf410	2	48	-0.6816	-0.2759	-0.3103	0.1159	-0.2113
f-48-lxf511	2	48	-0.3931	-0.1204	-0.3451	0.1395	-0.0670
f-48-lxf414	2	48	-0.4976	-0.0759	-0.1801	-0.0150	-0.2266
f-49-lxf401	2	49	-0.5035	-0.1773	-0.3357	0.1380	-0.1286
f-49-lxf429	2	49	-0.7614	-0.0223	-0.1782	0.1873	-0.7483
f-49-lxf505	2	49	-0.4899	-0.1501	-0.2256	0.1211	-0.2353
f-49-lxf404	2	49	-0.7049	-0.3158	-0.3500	0.2196	-0.2588
f-49-lxf419	2	49	-0.4791	-0.0929	-0.3016	0.0928	-0.1773
f-50-lxf515	2	50	-0.5186	-0.1083	-0.2225	0.0447	-0.2325
f-50-lxf517	2	50	-0.6181	-0.1441	-0.2455	0.1204	-0.3490
f-50-lxf514	2	50	-0.5623	-0.0812	-0.2748	0.1310	-0.3373
f-50-lxf524	2	50	-0.4179	-0.0961	-0.3304	0.0575	-0.0489
f-50-lxf510	2	50	-0.6166	-0.1235	-0.3200	0.1441	-0.3172
f-50-lxf530	2	50	-0.4845	-0.1239	-0.2185	0.0976	-0.2396
f-51-lxf531	2	51	-0.3445	-0.1377	-0.3147	0.1070	0.0010
f-51-lxm523	2	51	-0.5805	-0.0774	-0.3158	0.1144	-0.3017
f-51-lxf509	2	51	-0.4938	-0.1498	-0.2854	0.0639	-0.1225
f-51-lxf516	2	51	-0.5403	-0.1800	-0.3126	0.1057	-0.1534
f-51-lxf520	2	51	-0.3871	-0.0968	-0.4516	0.1129	0.0484
f-52-lxf507	2	52	-0.5121	-0.1509	-0.3857	0.0924	-0.0680
f-52-lxf527	2	52	-0.3935	-0.2198	-0.2731	0.1006	-0.0012
f-52-lxf519	2	52	-0.6419	-0.1070	-0.1832	0.1089	-0.4606
f-52-lxf506	2	52	-0.5744	-0.2303	-0.2734	0.2019	-0.2726
f-53-lxf503	2	53	-0.8818	-0.3859	-0.2327	0.1886	-0.4518
f-53-lxf504	2	53	-0.5658	-0.2010	-0.2200	0.1075	-0.2523
f-53-lxf502	2	53	-0.5878	-0.0308	-0.2896	0.1986	-0.4660
f-53-lxf522	2	53	-0.3791	-0.1467	-0.3502	-0.0253	0.1431
f-53-lxf518	2	53	-0.4613	-0.1646	-0.3128	0.2036	-0.1875

f-54-lxf501	2	54	-0.5260	-0.1129	-0.3213	0.0993	-0.1911
f-54-lxf529	2	54	-0.5377	-0.1196	-0.3757	0.1253	-0.1676
f-54-lxf532	2	54	-0.2097	-0.0968	-0.4032	0.0968	0.1935
f-54-lxf526	2	54	-0.4058	-0.2683	-0.4058	0.0469	0.2214
f-56-lxf513	2	56	-0.5668	-0.2943	-0.3079	0.1144	-0.0790
f-57-lxf528	2	57	-0.4928	-0.2106	-0.3572	0.1575	-0.0825
f-58-lxf525	2	58	-0.5985	-0.1723	-0.3904	0.1577	-0.1935
f-60-lxf621	2	60	-0.5288	-0.0937	-0.2403	0.0256	-0.2205
f-60-lxf605	2	60	-0.1820	-0.1403	-0.5158	0.0544	0.4197
f-60-lxf606	2	60	-0.4381	-0.1720	-0.3757	0.1276	-0.0181
f-61-lxf602	2	61	-0.6025	-0.1556	-0.1987	0.0422	-0.2904
f-61-lxf604	2	61	-0.7659	-0.0739	-0.1956	0.1157	-0.6121
f-61-lxf622	2	61	-0.4038	-0.1000	-0.2761	0.0711	-0.0988
f-61-lxf615	2	61	-0.6849	-0.1268	-0.2656	0.1039	-0.3964
f-61-lxf612	2	61	-0.5013	-0.2305	-0.2499	0.1897	-0.2106
f-62-lxf619	2	62	-0.2419	-0.2903	-0.5484	0.1290	0.4677
f-62-lxf610	2	62	-0.7387	-0.4871	-0.6890	0.1078	0.3296
f-63-lxf613	2	63	-0.4793	-0.2880	-0.3828	0.1151	0.0765
f-63-lxf629	2	63	-0.5590	-0.0631	-0.3267	0.2400	-0.4092
f-63-lxf617	2	63	-0.5299	-0.2090	-0.2886	0.2529	-0.2853
f-64-lxf603	2	64	-0.5508	-0.1324	-0.3657	0.1718	-0.2245
f-64-lxf614	2	64	-0.1296	-0.1481	-0.4630	0.0370	0.4444
f-64-lxf618	2	64	-0.6632	-0.1272	-0.2933	0.1160	-0.3588
f-65-lxf630	2	65	-0.7745	-0.1670	-0.2758	0.1540	-0.4857
f-65-lxf625	2	65	-0.4495	-0.1655	-0.4203	0.0561	0.0803
f-65-lxf607	2	65	-0.4398	-0.0516	-0.2910	0.0801	-0.1773
f-65-lxf608	2	65	-0.2245	-0.1662	-0.3848	0.0525	0.2741
f-65-lxf609	2	65	-0.4533	-0.1033	-0.3200	0.0690	-0.0991
f-66-lxf628	2	66	-0.6081	-0.1726	-0.2657	0.0527	-0.2225
f-66-lxf616	2	66	-0.5815	-0.1753	-0.2369	0.0911	-0.2605
f-66-lxf611	2	66	-0.4412	-0.2424	-0.3955	0.1185	0.0781
f-67-lxf620	2	67	-0.4286	0.0508	-0.5873	0.2524	-0.1444
f-67-lxf626	2	67	-0.2813	-0.1253	-0.4063	0.1094	0.1409
f-67-lxf624	2	67	-0.3492	-0.1984	-0.4603	0.0794	0.2302
f-67-lxf601	2	67	-0.4370	-0.1346	-0.3881	0.1088	-0.0230
f-68-lxf623	2	68	-0.6042	-0.0806	-0.3296	0.0845	-0.2786
f-68-lxf627	2	68	-0.3624	-0.1971	-0.2534	0.0617	0.0264
